# 20 years of model curricula in German-speaking countries

**DOI:** 10.3205/zma001273

**Published:** 2019-10-15

**Authors:** Claudia Kiessling, Thomas Rotthoff, Kai P. Schnabel, Christoph Stosch, Jutta Begenau

**Affiliations:** 1 Uni Witten/Herdecke, Fakultät für Gesundheit, Lehrstuhl für die Ausbildung personaler und interpersonaler Kompetenzen im Gesundheitswesen, Witten, Germany; 2 Universität Augsburg, Med. Fakultät, Lehrstuhl für Medizindidaktik und Ausbildungsforschung, Augsburg, Germany; 3 Universität Bern, Institut für medizinische Lehre, Abteilung für Unterricht und Medien, Bern, Switzerland; 4 Universität zu Köln, Med. Fakultät, Studiendekanat, Köln, Germany; 5 Charité – Universitätsmedizin Berlin, Berlin, Germany

## Introduction

"*The medical profession is of curious nature; time and again, brilliant minds have been pondering what is most essential aspect in this blend of science, art, craft, charitable activity, and business."* [H. Kerschensteiner 1920, nach [1]]

20 years ago, in 1999, the so-called model clause, then §36a [2], was incorporated in the Licensing Regulations for Doctors in Germany for the first time. Following this regulation, the Reformed Curriculum in Undergraduate Medical Education, which had been prepared for a long time, was launched at Charité-Universitätsmedizin Berlin in the same year. Subsequently, several model curricula as well as reformed traditional curricula and hybrid curricula were set up that proved so successful that the Science Council evaluated the model curricula as follows: “With their integrated curricula and their flexibility and willingness to accommodate necessary adjustments, model curricula promoted ongoing optimisation of medical studies in Germany.” [[3], p.10] At the same time, the Bologna Process, which was likewise initiated in 1999, triggered important new ideas for medical training in the German-speaking countries. There, the Bologna-compliant transformation has contributed to comprehensive reforms of the existing medical degree programmes, particularly in Switzerland.

After 20 years of the Model Clause and the Bologna Process, we have compiled this special issue to enable everyone to read about and critically assess the experiences of the existing model degree programmes, as well as exemplary reforms in traditional curricula, in 17 articles. Fifteen articles report on Germany and two on Switzerland. First, two articles introduce the Swiss curricula. They are followed by the German model curricula resp. Jena as a reformed traditional curriculum, with the articles being listed in chronological order based on the inception of the curriculum or reform. While the authors were asked to maintain a clear focus, it is often impossible to fully describe a process and the complexity of a curriculum, which often lasted years, on a few pages. Accordingly, the editors decided that the following aspects were to be highlighted: founding principle, organisation and structural entrenchment, teaching/learning culture, objectives, contents, didactics, assessment and evaluation. Within this framework, the authors have naturally defined their own core themes. Three author groups focused on specific aspects in their articles: on the internal medicine curriculum at the Hannover Medical School [4], the final year in Mannheim [5], and the scientific paper at the Charité [6]. One article outlines as a personal comment the perspective of an anatomical teaching coordinator in the transition to a modularly organised model study course [7]. 

In the first part of this editorial, we would like to provide an overview of the reform developments in medical education over the last 50 years in German-speaking countries by placing them into the historical context. In the second part, we give an overview of the curricula described in this special issue. In the third part, we use the previously outlined experiences to draw a conclusion as to what we find worth considering for future reform discussions and for shaping the training of medical students in future.

## 1. The reform of medical education in the German-speaking countries

In March 2017, the then Federal Minister of Health, Hermann Gröhe, and the then Federal Minister of Research, Johanna Wanka, as well as representatives of the Conference of Health and Education Ministers of the Länder and the Coalition Fractions of the German Bundestag published the “Master Plan Medical Studies 2020”. With it, “the course is to be set for the education of the next generations of doctors, who can live up to the challenges of a society of longer life” [[8], p.1]. The master plan announces 37 measures to change the study structure and training content, e.g. competence-oriented training in accordance with the national competence-based catalogue of objectives (NKLM) published in 2015 [http://www.nklm.de], practice-oriented training, stronger focus on general practice in training, and practice-oriented examinations. Other measures concern the admission to higher education and recruiting junior physicians for “nationwide GP care” [8]. The master plan also refers specifically to the model clause: “In view of the measures implemented in accordance with the master plan, the previous model clause will be reviewed and, if necessary, redesigned in order to optimise medical training [[8], item 11]. Item 4, Practice-oriented Examinations, stipulates: “By implementing standardised state examinations designed by the IMPP, we simultaneously eliminate differences between the current regular and model curricula and restore the comparability of performance standards at different faculties” [[8], item 4]. The measures outlined in the Master Plan are not new. Both German and international reform discussions in the last 50 years are worth a closer look – first and foremost the early 1990s, as they can be regarded as essential for the development of the model clause and the optimisation of medical studies in Germany resulting thereof. 

### Reform discussion of the seventies and eighties

An important milestone in the recent reform history in Germany was the introduction of the Licensing Regulations for Doctors in October 1970 [9]. The innovations concerned contents and subjects (new ones included occupational and social medicine, medical psychology, medical sociology, psychosomatics and psychotherapy) and a newly introduced central written examination modelled on the US-American multiple-choice system. The latter was the step towards a more objective examination procedure and led to the establishment of the Institute for Medical and Pharmaceutical Assessment Questions (Institut für Medizinische und Pharmazeutische Prüfungsfragen, IMPP). Even though the licensing regulations called for the integration of subjects and offered a great deal of leeway [10], these were hardly used by the faculties. University lecturers criticised that they no longer held direct examination competence, while students criticised the school-like character and atomisation of their degree programmes [11]. 

As early as 1969, the Science Council recommended introducing the block-release system in medical training, reducing the number of students in courses and focusing more strongly on bedside teaching. Cooperation between preclinical and clinical disciplines was to be fostered. It was also recommended to counter the danger of “over-specialisation” in medicine with integrating measures [12]. The Science Council thus joined the international discussion. In the 1950s, Case Western Reserve University switched its medical studies to organ-specific blocks in order to facilitate the integration of subjects and by thus integrated learning early on in the degree programme.

According to Norman [13], this subject integration constituted a counter-movement to the trend that began in the USA in the post-war period, where a large number of chairs of natural science were incorporated at medical departments, following substantial financial support by the National Institutes of Health to bolster scientifically founded achievements in medicine. As a result, preclinical subjects were considerably strengthened. As teachers were usually natural scientists without clinical experience, the biomedical basis was rarely linked to clinical applications [13].

Subject integration was improved through the introduction of problem-based learning (PBL) at McMaster University in Ontario, Canada. As a curricular concept, it adopted other learning theory approaches discussed at the time: self-directed learning, self-regulation, small group learning, fewer lectures and examinations. In Europe, Maastricht was the first to adopt the PBL concept, followed by other faculties. In Germany, the University of Witten/Herdecke, founded in 1982, became the first private German university with the aim of “testing and implementing new educational policy models in higher education” [[14], p.67]. Reform projects were also established in other towns (e.g. in Ulm, Hannover, Münster, Frankfurt am Main), focusing on different aspects and with different life spans [15].

The seventies also saw changes in Switzerland. In 1970/1, a new medical study regulation was issued in Bern. It made innovations possible that had already been discussed on an international level for several years: shortening of the pre-clinical period, massive reduction of lectures in favour of group and block-release training, introduction of an elective study year. As early as 1961, Hannes Pauli and other senior physicians founded the “group of young angry men” (referred to by insiders as “Wurst-Club”, i.e. “sausage club”), who initiated a fundamental reform of medical education and training together with Ettore Rossi [16]. 

In the following years, various groups submitted recommendations for a reform of medical education, which were partly reflected in various amendments to the licensing regulations in Germany, e.g. in 1989 a stronger focus on patient orientation during the pre-clinical phase. A recommendation by the German Science Council in 1982 cited the inadequate training of practical skills and the inadequate acquisition of medical decision-making skills as shortcomings of the medical education at the time. The Science Council saw the reason for this in, for example, the increased number of students, while the number of beds in university clinics and medical staff remained the same. To redress the problem, the Science Council recommended the following: fostering practical, patient-oriented training, involvement of non-university hospitals and clarification of the Capacity Regulations, which have been frequently revised and are still in force today [17]. Additional reform proposals concerned the introduction of electives to reduce the number of required subjects, and the assessment system. An explicit curricular incorporation of scientific activities in medical studies had not played a major role in the recommendations until then; moreover, since 1990 it was no longer necessary to complete a medical diploma thesis, as had been required for medical degrees in the GDR, without any equivalent format being introduced [10]. Since Frunder & Machnik [18] presented a detailed description of the study reforms in the GDR in 1993, it is not necessary to reiterate it at this point.

The nationwide student strike in 1988/89 introduced new momentum to the debate, with students voicing vehement demands for a reform of medical studies. Following the fall of the Berlin Wall in 1989, the discussions on medical education in East and West were consolidated.

#### Reform discussion of the nineties 

In 1992, the Science Council published a comprehensive document with guidelines for the reform of medical studies [19] in which it stated that medical education was no longer in a position to adequately integrate the scientific, technical and social changes as well as the manifold expectations placed on doctors into the degree programme. Accordingly, it advocated a “paradigm shift” in medical education: “it is time to say goodbye to the image of the physician who can do everything alone” [[20], p.52]. In 1989 and 1995 respectively, the Murrhardter Kreis, a group of experts supported by the Robert Bosch Foundation, published “The Future Doctor”, an analysis of future demands on doctors, the consequences for training and ways to reform them [19]. From today’s perspective and the authors’ point of view, both publications can be described as groundbreaking for further reform discussions in Germany. Table 1 (Tab. 1) shows the most important recommendations issued by the Science Council and Murrhardter Kreis.

Unlike the Science Council, the Murrhardter Kreis joined the international discussions on strengthening so-called “community-oriented education”, the aim of which was to align itself to the needs of the population resp. community and to strengthen the health of the population and primary care [21], [22]. 

Numerous reform ideas were also adopted by the German Medical Faculty Association in 1996 [23]. The required fundamental reorganisation of medical studies (at least for the model curricula) was eventually facilitated in a first step by Andrea Fischer, then Federal Minister of Health. She issued the Eighth Regulation amending the Licensing Regulations in 1999, the essential amendment of which made the admission of a model curriculum possible as per §36a [2]. 

Likewise in 1999, the Bologna Process commenced, which paved the way for the development of a coherent European Higher Education Area [24]. While Germany was extremely sceptical about adapting medical studies to Bologna guidelines, other countries, including Switzerland, rose to the challenge and converted medical studies to a Bachelor’s/Master’s degree programme [25]. Simultaneously, the international debate on learning objectives as a steering instrument for curriculum development led to the development of the Swiss Catalogue of Learning Objectives (SCLO) [26]. A pilot accreditation of the medical faculties had previously identified the need for reform and led to a number of reforms [27], [28]. 

#### Reform discussion of the 2000s 

In Germany, a further reform step was made possible by the amendment of the Licensing Regulations 2002, which came into force in October 2003. Here, the regulation of model curricula as per §41 was implemented and, above all, a number of innovations were introduced for traditional curricula, even if these were very similar to a ministerial draft bill that had been submitted in 1995 and were therefore not entirely new [29]. These included, among other things: cutting down state examinations from four to two and increasing the number of graded examinations throughout the degree programme; a better integration of pre-clinical and clinical subjects, especially in the pre-clinical phase; the introduction of cross-sectional areas and interdisciplinary assessments to promote interdisciplinary teaching; a stronger focus on practical training; and strengthening of general practice. The introduction of graded subjects meant that almost 50 compulsory subjects had to be examined. And early on, people feared a significant increase in factual knowledge, a breaking-down and school-like conditions [30]. However, the pre-clinical and clinical phases were still separated by the preclinical medical examination (Physikum), and an additional side effect was the abolition of the physician-in-training phase (Arzt im Praktikum) in 2004.

A comparison of the various recommendations of the last 50 years and the current recommendations in the Master Plan 2020 reveals certain parallels regarding themes and recommendations. A dispute appears to be going on over the number of state examinations as a control instrument of the state over the universities on the one hand, and the preservation of the academic freedom of the universities on the other. The supposed dichotomy between practical training and the scientific foundation of medical studies, as well as a possible integration of disciplines versus traditional discipline-specific nature of the course also appear to be recurring themes. 

Recommendations and changes have, moreover, always been affected by political factors. The evidence of teaching-learning arrangements or assessment formats usually played a secondary role. Numerous recommendations, in particular the vertical integration of subjects, the modularisation of degree programmes, the establishment of individual priorities, student-centred learning and greater practical relevance were specifically implemented in the model curricula, as noted by the Science Council in 2014 [3]. Some of these recommendations are today an integral part of the Master Plan 2020. 

## 2. Overview of the curricula presented in this special edition

This special issue features a compilation of almost all model curricula in Germany. Table 2 (Tab. 2) provides a complete overview of the model curricula that have been established in Germany since 1999.

In addition to the model curricula listed above, the Medical Faculty of Jena is also featured in this special issue with a reformed traditional curriculum, in which inclination-oriented courses totalling 21 semester hours per week (SWS) have been available to students as elective subjects in the clinical section since winter semester 2012/13 [31]. 

### Foundation histories of model curricula in Germany 

As an introduction to this special issue, we would like to provide a brief overview of the degree programmes’ foundation histories, aiming to inspire readers to read up on the structure of the individual degree programmes with regard to objectives, contents and methods in the individual articles.

There are many reasons why universities decided to establish a model curriculum in accordance with §42. Witten/Herdecke aimed to establish “interdisciplinary, longitudinal formats” and to implement “equivalent examination formats to replace state examinations” [32]. Brandenburg Medical School, likewise a private medical school, saw an opportunity for a PBL-based and competence-oriented curriculum and for integrated interdisciplinary modules that “consolidate basic, clinical-theoretical and clinical subjects right from the start” [33]. For most state-funded medical faculties, it was a mixture of objective reasons, internal changes and external impulses. In Aachen, for example, a negative evaluation by the Science Council resulted in the initiation of a new a model curriculum that “facilitated a reorientation of medical education” [34]. In Bochum, the faculty administration together with the students decided to press ahead with the study reforms “with regard to the future prospects of medical education in NRW” [35]. Following the reform in the 1990s, the curriculum in Cologne had already mapped the essential structural building blocks of the new Licensing Regulations for Doctors [30], and the University of Cologne intended to subsequently expand its scope with a model curriculum. As part of the process, the university planned to reduce the “discrepancy between study-related qualification and required professional qualification” and to counteract the increasing “shortage of doctors in clinical curative medicine and primary care” [36]. In Hannover, the dean’s office initiated a working group “which, within the framework of the study commission, will further develop its own ideas, in order to implement patient-oriented training that is more closely geared to medical competencies” [37]. Capacity-relevant considerations also played a role.

In Hamburg, the starting point were a graduate survey and the awareness of the Bologna Process. For this reason, “lecturers from all disciplines at the Medical Faculty at the University of Hamburg and the University Medical Center Hamburg-Eppendorf” began to develop the model curriculum “together with students in a comprehensive work and coordination process” [38]. Mannheim was faced with the task of expanding medical education and strengthening “basic research and teaching” [5], and Oldenburg was to see the establishment of a medical faculty. This was underpinned by close cooperation with Groningen University and its competence-oriented degree programme [39].

The roots of the Berlin reformed curriculum were of a different kind [40], as it had been initiated by students. Their dissatisfaction with the study conditions and participation options had led to a strike in 1989 that later spread across the whole country. This bottom-up genesis is certainly a possible explanation for the to some extent vehement and long-lasting intrafaculty resistance. Moreover, there was little potential for reconciliation in the 10 years of parallel existence of the reformed curriculum and the traditional curriculum. The model curriculum established in 2010, on the other hand, drew on the study and assessment modalities of the reformed curriculum as well as on teaching experience – as required by the Land; like at most other universities, it was initiated by the dean’s office for student affairs [40], [41].

In Bochum and Hamburg, too, the traditional and reformed curricula initially existed side by side. In Bochum, the model and traditional curricula were combined to form an integrated reformed curriculum in the sense of a reformed traditional curriculum. The termination of the first model curriculum in Hamburg in 2001 was justified as follows: “The faculty could neither do justice to the model curriculum nor to the traditional curriculum. That’s because three different curricula existed following the introduction of the new Licensing Regulations for Doctors: the old and the new licensing regulations as well as the model curriculum” [42]. In other words, the three model curricula that had been developed next to the traditional curricula no longer exist in their original form.

#### Founding principles and aims of the model curricula

Moreover, the universities pursued different founding principles. In Witten/Herdecke, for example, the aim was to set up a free university that was largely independent of state regulations, and to “establish culturally effective institutions in the sense of a liberal intellectual life” [32]. A major incentive for the establishment of the latest model curriculum in Germany, namely in Brandenburg, was to “train more doctors for the non-metropolitan region, thus improving medical care in rural regions” [33]. It is noteworthy that the Brandenburg team of authors also includes students, i.e. that student participation in the reform process was also reflected in the authorship of the article.

Jena strived to provide students with more inclination-oriented practical approach in their degree programme and set up a comprehensive clinical elective. Mannheim used the model clause primarily to subdivide the final year (Praktisches Jahr) into quarters. The aim was to strengthen the focus on outpatient medicine in medical education. Unlike all other model curricula, Mannheim retained the preclinical medical examination (Physikum) [5]. Strengthening the relevance of outpatient care also played an important role in Cologne [36]. Oldenburg sought ways from the outset to establish a collaboration with the University of Groningen and to transfer central elements of the competence-based medical studies in Groningen into the German context [39]. Oldenburg and Hamburg refer explicitly to the opportunities and possibilities of the Bologna Process. Because of these different objectives, the respective degree courses were structured in different ways, too.

#### Medical education reforms in Switzerland

In Switzerland, the dissatisfaction of lecturers and students with the study conditions and a discussion in the professional societies at the end of the 1980s triggered relevant reform ideas for the faculties. The Federal Government agreed to approve so-called derogations (similar to the model clause in Germany) to the valid ordinance by the Federal Office of Public Health. A pilot accreditation by the medical faculties in 1999 supported and stabilised the process and resulted in all five medical faculties (Basel, Bern, Lausanne, Geneva and Zurich) submitting such derogations by 2002, which were subsequently approved. In Switzerland, this granted the medical faculties more liberties, which were, however, subject to outcome-based control by a common catalogue of learning objectives with a final state examination. For this purpose, a binding catalogue of learning objectives was drawn up by the Swiss Medical Interfaculty Commission (SMIFK), which has since then constituted the binding examination basis in Switzerland [27]. In Bern, this initiative found a faculty willing to reform that had already had a lot of experience with innovative forms of teaching through the introduction of small group teaching and block internships at the bedside in 1973. In Geneva, the curriculum was likewise converted into an integrated concept with problem-based learning and communication training in 1995. In Zurich, a core curriculum has been set up, where students have the option to choose individual priorities in various tracks at an early stage. In Fribourg, the youngest of the six Swiss medical faculties that offer a complete Bachelor’s and Master’s degree programme, 40 students specialising in “general practice” and "social responsibility" will be training this year in accordance with a consistent “programmatic assessment design” [27]. Switzerland’s oldest medical faculty in Basel introduced an organ system-based hybrid curriculum structure with PBL units and OSCE, complemented by so-called competence tracks in 1998 [28]. All degree programmes in Switzerland have in common that they are based on a consecutive Bachelor’s-Master’s programme that prepares students for a consistent federal theoretical and practical final state examination, which is defined by the new central national catalogue of learning objectives called “PROFILES”. [43], [44]

## 3. Conclusion and recommendations for the future

### Complex change processes are challenging

The model curricula, but also many traditional curricula, are struggling to implement central recommendations for the reform of medical studies that have been drafted for decades. The question is why key recommendations such as those called for 30 years ago by the Murrhardter Kreis and the Science Council, e.g. downscaling the core curriculum, opportunities to focus on specific areas, vertical subject integration and strengthening outpatient care, are seemingly so difficult to implement. The licensing regulations of 1970 and 2003 apparently did not lead to these key recommendations being sustainably initiated at all universities. According to the Science Council in 2014, however, the model clause was a successful engine for reforms. The counter-argument here is the fact that the first model curricula have already been discontinued or transformed into hybrid curricula and that the Master Plan 2020 remains vague on the future handling of model curricula. 

Why is sustainable change so difficult? What are the barriers that oppose change? Dieter Scheffer, Dean at the Charité of many years and co-founder of the Berlin Reformed Medical Curriculum, wrote in 1999: “Despite all changes, however, an old demand went unheard (…) “Teachers teach less so that students can learn more” (Comenius 1640)” [[45], p.10]. He follows up this statement with three essential reasons: “lacking realisation that a fundamental reform of the academic system is required; holding on to the traditional self-image of university teachers; an incomplete conception of man and of the profession on the part of physicians.” [45][, p.10]

In 2015/2016, Velthuis and colleagues conducted interviews with individuals who had initiated change processes at medical faculties in the Netherlands. They identified three main difficulties: the large number of people involved and affected with a multitude of different perspectives, and the questions of how to deal with resistance and how to manage the change process [46]. The authors of this special issue outline additional challenges, including questions of financing, capacities and student numbers. Many universities consider the transition to smaller groups in the classroom as well as vertical integration of subjects to constitute a challenge for learning culture, logistics and capacity [7], [35].

Commenting on the new Licensing Regulations from 1970, Thure von Uexküll pointed out in 1971 that the medicine of 1971 would no longer be the medicine of the year 2000, and that medical faculties would have to constantly adapt their curricula to ongoing developments in the field of medicine: “In order to achieve all this, medical faculties will have to be extremely flexible in future. This, however, is hindered by the fact that they are tied up in a complex web of dependencies between law and regulation authorities and ministries which decide on budget and staffing plans.” [[47], p.712]

Does this mean that we need a new mode of discussion about reforming medical education? And what could that achieve? In all the projects presented, the management of change and the development of competence and structures played a decisive role. In many projects, these new structures are managed top-down but to a large extent participatively (e.g. by the dean’s office, dean’s office for student affairs or reform officers). New commissions and working groups have been established, deans’ offices for student affairs enhanced, faculty development training courses established, closed-door meetings held. All this has paved the way for the involvement of the entire faculty in the development process – clinical and non-clinical faculty, students and planners. As teaching attained greater visibility, it gained a higher status, e.g. in Cologne [36]. In Bochum, a “shared culture of exchange” emerged [35].

Hamburg created a new incentive and reward system with a variety of measures to support teaching. At many universities, systematic evaluation played a crucial role in optimising processes and highlighting strengths and weaknesses. Hannover introduced regular graduate surveys and, in addition to student surveys such as were deployed at many universities, a teacher survey [37]. At some universities, reform processes were considerably boosted by technical solutions, e.g. assessment databases, content management and e-learning platforms or student information systems.

So perhaps what we need is not so much standardised comparability and state regulation (e.g. through a large number of state examinations), but rather more exchange, scientific discourse, mutual support and the courage to embrace change. What should be our guiding principle for implementing innovations? The “Science for Future” movement put it this way: “What do we want? Evidence-based science. When do we want it? After peer review!” We should adopt this demand with regard to the reform of medical education. Key recommendations must also undergo an evidence-based appraisal. An interesting example would be a critical evaluation of the currently favoured competence orientation in medical studies (as well) [48].

A medicine of the future will result in new demands on the medical profession. Technologisation and the economisation of the healthcare system play an essential role. In a medical world that is tightly packed and geared towards profitable operations, patients want time and to be heard. So how can a medicine be maintained in the future that was once defined by Uexküll as the “way people interact with people”? [49]. 

We hope that in future, too, model curricula will help students listen to patients’ voices during their degree courses and teach medical students to become competent and satisfied doctors of the future. From our point of view – after reading this special issue – they do have considerable potential.

## Acknowledgements

The authors would like to thank the Robert Bosch Foundation for its financial support for this special issue. We would also like to thank the German Association for Medical Education for incorporating this special issue into the GMS JME. In particular, we thank Beate Hespelein for her great commitment to prepare this special issue. We would also like to thank the authors and reviewers without whom this special issue would not have been possible. Many thanks also to our co-editors Johann Arias and Melanie Simon (both Aachen).

## Competing interests

The authors declare that they have no competing interests. 

## Figures and Tables

**Table 1:**
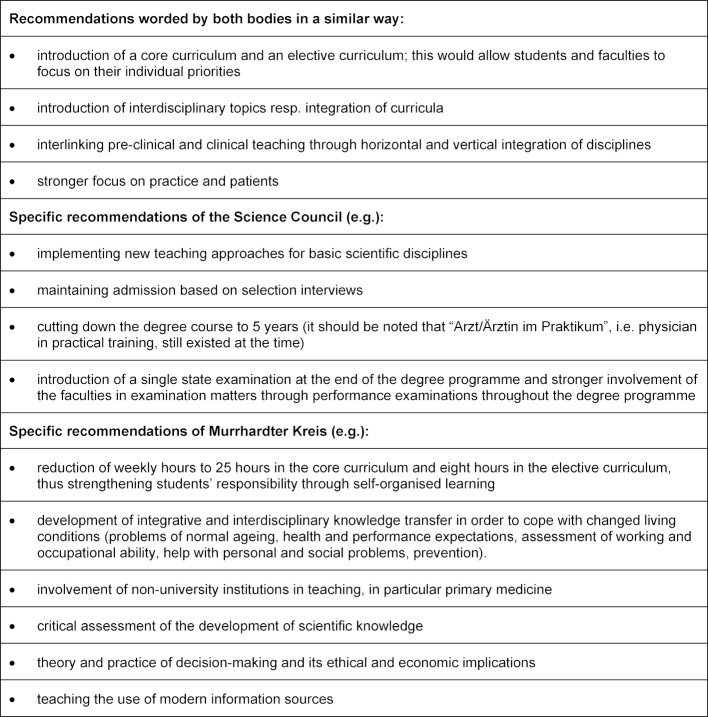
Recommendations of the Science Council 1992 and Murrhardter Kreis 1995

**Table 2:**
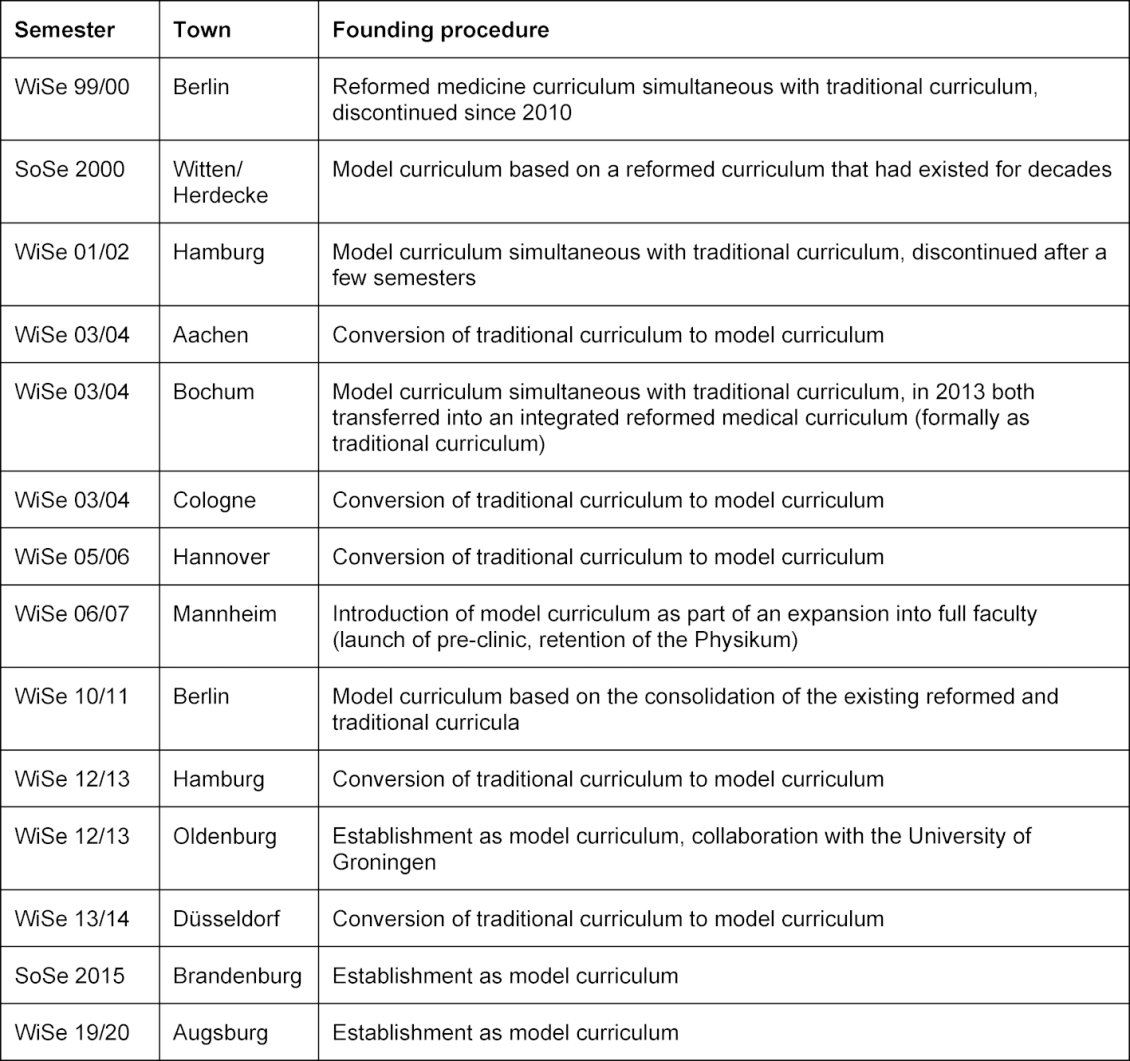
Model curricula in Germany since 1999

## References

[1] von Salis Soglio G (2016). Medizinsicher Ethos: Brauchen wir ein neues Arztbild. Dtsch Arztebl.

[2] Bundesministerium für Bildung und Gesundheit (1999). Achte Verordnung zur Änderung der Approbationsordnung für Ärzte. Bundesgesetzbl.

[3] Wissenschaftsrat (2014). Empfehlungen zur Weiterentwicklung des Medizinstudiums in Deutschland auf Grundlage einer Bestandsaufnahme der humanmedizinischen Modellstudiengänge.

[4] Bintaro P, Schneidewind S, Fischer V (2019). The Development of the Internal Medicine Courses at Hannover Medical School from 2001 to 2018. GMS J Med Educ.

[5] Liebke L, Narciß E, Obertacke U, Fritz-Joas F (2019). Restructuring the final year of the model study programme MaReCuM at the medical faculty Mannheim: the academic quarter in ambulatory medicine. GMS J Med Educ.

[6] Drees S, Schmitzberger, Grohmann G, Peters H (2019). The scientific term paper at the Charité: a project report on concept, implementation, and students' evaluation and learning. GMS J Med Educ.

[7] Winkelmann A (2019). A teaching coordinator's nightmare?. GMS J Med Educ.

[8] Bundesministerium für Bildung und Forschung (2017). Masterplan Medizinstudium 2020.

[9] Bundesministerium für Bildung und Gesundheitheit (1970). Approbationsordnung für Ärzte. Bundesgesetzbl.

[10] Gulich MS (1999). Medizinische Ausbildung: Irrtümer – und kein Ende?. Dtsch Arztebl.

[11] Schagen U, Habeck D, Schagen U, Wagner G (1993). Rahmenbedingungen der Studienreform an west- und ostdeutschen Universitäten. Reform der Ärzteausbildung: neue Wege in den Fakultäten.

[12] Wissenschaftsrat (1968). Empfehlungen des Wissenschaftsrats zur Struktur und zum Ausbau der medizinischen Forschungs- und Ausbildungsstätten.

[13] Norman G (2012). Medical education: past, present and future. Perspec Med Educ.

[14] Butzlaff M, Hofmann M Der Modellstudiengang Medizin an der Universität Witten/Herdecke – auf dem Weg zur lebenslang lernfähigen Arztpersönlichkeit. Methoden und Verfahren des Qualitätsmanagements.Praxisbeispiele und Innovationen. Unterkapitel E8.

[15] Habeck D, Schagen U, Wagner G (1993). Reform der Ärzteausbildung: neue Wege in den Fakultäten.

[16] Steiger J (2004). In memoriam. Prof. Dr. med. Hannes G. Pauli (1924–2003). Ein grosser Pionier der Ärzteausbildung. Schwsch Ärztez.

[17] Wissenschaftsrat (1982). Stellungnahme zu Fragen der ärztlichen Ausbildung.

[18] Frunder H, Machnik G, Habeck D, Schagen U, Wagner G (1993). Die Reformdiskussion in der DDR. Reform der Ärzteausbildung: neue Wege in den Fakultäten.

[19] Murrhardter Kreis (1995). Das Arztbild der Zukunft. Analysen künftiger Anforderungen an den Arzt. Konsequenzen für die Ausbildung und Reform zu ihrer Reform.

[20] Wissenschaftsrat (1992). Leitlinien zur Reform des Medizinstudiums.

[21] Hamad B (1991). Community-oriented medical education: what is it?. Med Educ.

[22] Schmidt HG, Neufeld VR, Nooman ZM, Ogunbode T (1991). Network of community-oriented educational institutions for the health sciences. Acad Med.

[23] Kirchner T (2002). Die neue Ärztliche Approbationsordnung: Bedeutung für die ärztliche Ausbildung.

[24] Harendza S, Guse AH (2009). Das Medizinstudium als Bachelor- und Master-Studiengang. Bundesgesundheitsbl Gesundheitsforsch Gesundheitsschutz.

[25] Kaiser HJ, Kiessling C (2010). Two-cycle curriculum - bachelor-master structure according to the Bologna agreement: the Swiss experience in Basle. GMS Z Med Ausbild.

[26] Burgi H, Rindlisbacher B, Bader C, Bloch R, Bosman F, Gasser C, Gerke W, Humair JP, Im Hof V, Kaiser H, Lefebvre D, Schläppi P, Sottas B, Spinas GA, Stuck AE (2008). Swiss Catalogue of Learning Objectives for Undergraduate Medical Training. Under a Mandate of the Joint Commission of the Swiss Medical Schools.

[27] Bonvin R, Nendaz M, Frey P, Schnabel K, Huwendiek S, Schirlo C (2019). Looking back: twenty years of reforming undergraduate medical training and curriculum frameworks in Switzerland. GMS J Med Educ.

[28] Voigt G, Wilde M (2019). Bologna backstage - experiences from behind the scenes of the reforms of the Basel medical curriculum. GMS J Med Educ.

[29] Stosch C, Lehmann K, Herzig S (2008,3(3)). Time for Change - Die Implementierung des Modellstudiengangs Humanmedizin in Köln. ZFHE.

[30] Schagen U (2002). Reformen auf dem Papier - Studium der Humanmedizin in der Bundesrepublik Deutschland seit 1970. Jahrbuch krit Med.

[31] Ehlers C, Wiesener N, Teichgräber U, Guntinas-Lichius O (2109). Reformed conventional curriculum promoting the professional interest orientation of students of medicine: JENOS. GMS J Med Eudc.

[32] Frost K, Edelhäuser F, Hofmann M, Tauschel D, Lutz G (2019). History and development of medical studies at the University of Witten/Herdecke – an example of "continuous reform". GMS J Med Educ.

[33] Winkelmann A, Schendzielorz J, Maske D, Arends P, Bohne C, Hölzer H, Harre K, Nübel J, Otto B, Oess S (2019). The Brandenburg reformed medical curriculum: study locally, work locally. GMS J Med Educ.

[34] Simon M, Martens A, Finsterer S, Sudmann S, Arias J (2019). The Aachen model study course in medicine – development and implementation fifteen years of a reformed medical curriculum at RWTH Aachen University. GMS J Med Educ.

[35] Burger A, Huenges B, Köster U, Thomas M, Woestmann B, Lieverscheidt H, Rusche HH, Schäfer T (2019). 15 years of the model study course in medicine at the Ruhr University Bochum. GMS J Med Educ.

[36] Zims H, Karay Y, Neugebauer P, Herzig S, Stosch C (2019). Fifteen years of the Cologne medical model study course: has the expectation of increasing student interest in general practice specialization been fulfilled?. GMS J Med Educ.

[37] Paulmann V, Fischer V, Just I (2019). HannibaL – the model curriculum at Hannover Medical School: targets, implementation and experiences. GMS J Med Educ.

[38] Rheingans A, Soulos A, Mohr S, Meyer J, Guse AH (2019). The Hamburg integrated medical degree program (iMED). GMS J Med Educ.

[39] Gelhar K (2019). The model medical degree programme "human medicine" in Oldenburg - the european medical school Oldenburg-Groningen. GMS J Med Educ.

[40] Begenau J, Kiessling C (2019). The Berlin reformed curriculum in undergraduate medical education: a retrospective of the development history, principles, and termination. GMS J Med Educ.

[41] Hitzblech T, Maaz A, Rollinger T, Ludwig S, Dettmer S, Wurl W, Roa-Romero Y, Raspe R, Petzold M, Breckwoldt J (2019). Peters H. The modular curriculum of medicine at the Charité Berlin – a project report based on an across-semester student evaluation. GMS J Med Educ.

[42] Hibbeler B (2006). Modellstudiengänge: Wissen, wofür man lernt. Dtsch Ärztebl Studieren.de.

[43] Michaud PA, Jucker-Kupper P, Profiles working group (2016). The "Profiles" document: a modern revision of the objectives of undergraduate medical studies in Switzerland. Swiss Med Wkly.

[44] Profiles A mandate of the Joint Commission of the Swiss Medical Schools.

[45] Scheffner D, Göbel E, Schnabel K (1999). Die Reform des Medizinstudiums – Bestreben und Bedenken. Medizinische Reformstudiengänge.

[46] Velthuis F, Varpio L, Helmich E, Dekker H, Jaarsma ADC (2018). Navigating the Complexities of Undergraduate Medical Curriculum Change: Change Leaders’ Perspectives. Acad Med.

[47] von Uexküll T (1971). Das Problem der Ausbildung zum Arzt in der modernen Welt. Dtsch Arztebl.

[48] Rotthoff T (2018). Standing up for Subjectivity in the Assessment of Competencies. GMS J Med Educ.

[49] von Uexkuell T, Wiese J (1990). Medizin als Umgang von Menschen mit Menschen. Psychosomatische Medizin in Kindheit und Adoleszenz.

